# Differential Time-of-Day Effects of Caffeine Capsule and Mouth Rinse on Physical Performance and Volleyball-Specific Skills in Adolescent Male Volleyball Players

**DOI:** 10.3390/nu18101514

**Published:** 2026-05-09

**Authors:** Salma Belhaj Amor, Wissem Dhahbi, Marouen Souaifi, Halil İbrahim Ceylan, Johnny Padulo, Stefano Vando, Nagihan Burçak Ceylan, Raul Ioan Muntean, Nizar Souissi

**Affiliations:** 1 Physical Activity, Sport and Health Research Unit (UR18JS01), National Observatory of Sports, Tunis 1003, Tunisia; salmabhamor@gmail.com (S.B.A.); n_souissi@yahoo.fr (N.S.); 2 High Institute of Sports and Physical Education of El Kef, University of Jendouba, El Kef 7100, Tunisia; 3 Research Unit (UR22JS01) “Sport Sciences, Health and Movement”, High Institute of Sport and Physical Education of Kef, University of Jendouba, El Kef 7100, Tunisia; wissem.dhahbi@gmail.com (W.D.); maru@gmx.fr (M.S.); 4 Training Department, Police College, Qatar Police Academy, Doha 7157, Qatar; 5 Physical Education of Sports Teaching Department, Faculty of Sports Sciences, Atatürk University, 25240 Erzurum, Türkiye; 6 Department of Biomedical Sciences for Health (SCIBIS), Università Degli Studi di Milano, 20134 Milan, Italy; johnny.padulo@unimi.it; 7 Fitkion—Center for Kinesiology and Adapted Physical Activity, 04100 Latina, Italy; stefanovando@gmail.com; 8 Graduate Education Institute, Bayburt University, 69000 Bayburt, Türkiye; burcaksehitoglu@gmail.com; 9 Department of Physical Education and Sport, Faculty of Law and Social Sciences, University “1 Decembrie 1918” of Alba Iulia, 510009 Alba Iulia, Romania; 10 High Institute of Sport and Physical Education, University of Manouba, Ksar-Said, Manouba 2010, Tunisia

**Keywords:** caffeine, mouth rinse, time of day, volleyball, jump performance, change-of-direction speed, skill accuracy, adolescent athletes

## Abstract

Objective: This study compared the acute effects of caffeine capsule ingestion and caffeine mouth rinsing on physical performance and volleyball-specific skills at different times of day in trained adolescent male volleyball players. Methods: Twenty-four well-trained male volleyball players (age: 16.9 ± 0.7 years) completed a randomized, double-blind, placebo-controlled crossover study involving three supplementation conditions, caffeine capsule (CAFcap, 3 mg·kg^−1^), caffeine mouth rinse (CAFrinse, 3 mg·kg^−1^), and placebo (PLA), administered via a double-dummy procedure (nine sessions per participant: 3 conditions × 3 times of day) at 08:00, 12:00, and 18:00. Participants completed squat jump (SJ), countermovement jump (CMJ), block jump, attack jump, 10 × 10 m *t*-test, spike accuracy, and serve accuracy assessments. Data were analyzed using two-way repeated-measures ANOVA. Results: Significant main effects of condition and time of day were observed for all outcomes. Significant condition × time-of-day interactions were found for SJ, CMJ, attack jump, and change-of-direction speed, indicating that caffeine-related ergogenic effects were most evident in the morning and at midday, whereas these benefits were attenuated in the evening when baseline performance was highest. At 08:00 and 12:00, both CAFcap and CAFrinse improved jump performance and agility compared with PLA, with capsule ingestion showing a small-to-moderate advantage over mouth rinsing for selected lower-limb power outcomes at midday (mean difference range: 0.51–0.57 cm; dz = 0.57–0.65). For block jump, spike accuracy, and serve accuracy, both caffeine conditions improved performance relative to placebo, while a progressive improvement across the day was observed under all conditions, including placebo, confirming a diurnal rhythm effect independent of supplementation. Overall, the data indicate that caffeine partially reduced the amplitude of diurnal variation in several physical performance measures. Conclusions: Both caffeine capsule ingestion and caffeine mouth rinsing enhanced physical and volleyball-specific performance in trained adolescent male volleyball players. The ergogenic effects were more pronounced earlier in the day, suggesting that caffeine may be particularly useful for attenuating morning and midday performance decrements, while mouth rinsing represents a practical non-ingestive alternative with meaningful efficacy.

## 1. Introduction

Volleyball is an intermittent, high-intensity sport that requires repeated explosive actions, such as jumps, rapid accelerations, and powerful spikes, all executed under substantial neuromuscular and cognitive demands [1,2,3]. These physical and technical requirements make performance highly sensitive to factors that influence power production, reaction speed, and precision [4]. Among ergogenic strategies used to optimize these determinants, caffeine supplementation remains one of the most widely adopted and scientifically supported.

Caffeine is consumed globally in multiple forms and has well-established ergogenic effects across endurance, strength, power, and skill-based tasks [5,6,7,8]. Its widespread use led to its temporary classification as a prohibited substance from 1984 to 2004 [9]; following removal from the WADA prohibited list, caffeine has remained on the Monitoring Program, reflecting sustained regulatory interest in its use at doses conferring substantial competitive advantages [9,10]. Current recommendations advise doses of 3–6 mg·kg^−1^ for adult athletes [11,12]. In adolescent populations, 3 mg·kg^−1^ represents the conservative lower bound, yielding moderate cognitive and physical improvements with a favorable tolerability profile, whereas higher doses disproportionately elevate adverse event rates in low-habitual consumers [10,13]. Although caffeine is commonly ingested in capsule or beverage form 45–60 min before exercise [14], alternative administration methods such as mouth rinsing have recently gained attention. By stimulating bitter taste receptors and orosensory–cortical pathways, caffeine mouth rinse may theoretically enhance central arousal without systemic absorption [15]. Yet, evidence supporting its efficacy remains inconsistent, with many trials reporting negligible effects [16,17] and systematic reviews highlighting strong protocol dependency [18,19]. Nevertheless, protocol-specific benefits have been reported for caffeine mouth rinsing on strength, muscular endurance, and cognitive performance, suggesting that orosensory stimulation may be sufficient to induce ergogenic effects under selected task conditions [20].

Capsule ingestion provides full systemic caffeine bioavailability, with plasma concentrations peaking approximately 45–60 min post-ingestion, but requires advance planning and may cause gastrointestinal discomfort in susceptible individuals [10,14]. Mouth rinsing permits immediate pre-exercise administration without ingestion, reduces gastrointestinal burden, and may be preferred when time or tolerance constraints preclude capsule use; however, its ergogenic effects appear protocol-dependent and less consistent [18,19]. The differential pharmacological profile of the two routes (full systemic exposure versus exclusively orosensory stimulation) provides a mechanistic rationale for expecting capsule ingestion to yield greater and more consistent ergogenic benefits across a broader range of performance outcomes [10,14].

Time-of-day (TOD) variation represents another key factor influencing athletic performance. Circadian rhythms modulate physiological, endocrine, and neurocognitive processes relevant to sport, producing well-documented fluctuations in strength, power, reaction time, and technical accuracy [21,22]. Performance commonly peaks in the late afternoon, whereas morning sessions are associated with reduced alertness, slower neuromuscular activation, and diminished explosive output [23,24]. Moreover, the magnitude of these diurnal variations appears to be influenced by circadian preference, as misalignment between testing time and an individual’s circadian rhythm can impair both cognitive and physical performance [25]. Importantly, caffeine appears capable of attenuating or reversing these morning decrements, particularly in youth athletes [26]. This suggests a potential interaction between circadian biology and caffeine responsiveness, yet this interaction remains insufficiently explored in adolescent team-sport athletes and virtually unknown in volleyball.

To the authors’ knowledge, no study has simultaneously compared caffeine ingestion and caffeine mouth rinsing across multiple daily timepoints while assessing both physical capacities and volleyball-specific skills. This represents a meaningful gap, given that adolescent volleyball players frequently train or compete at suboptimal circadian phases and may benefit from more practical or rapid caffeine-delivery methods.

Therefore, the present study investigated the acute effects of isodosed (3 mg·kg^−1^) caffeine capsules, caffeine mouth rinse, and placebo on physical performance and volleyball-specific skills at 08:00, 12:00, and 18:00 in trained adolescent male volleyball players. We hypothesized that (i) caffeine ingestion would yield superior ergogenic effects compared with mouth rinsing, and (ii) these effects would be most pronounced in the morning and midday, thereby reducing the amplitude of diurnal performance variation.

## 2. Methods

### 2.1. Study Design

**Figure 1:**
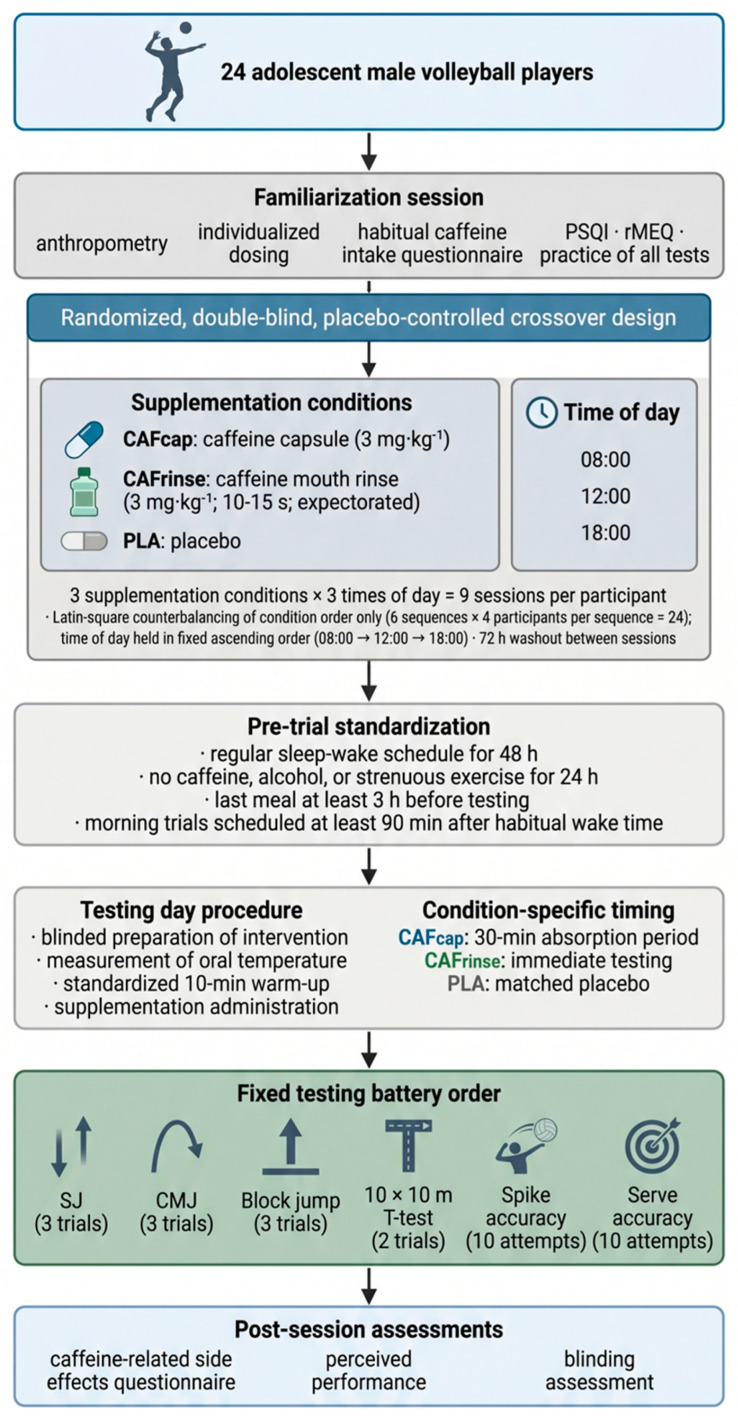
Flowchart of the randomized, double-blind, placebo-controlled crossover protocol investigating the effects of caffeine capsule, caffeine mouth rinse, and placebo at 08:00, 12:00, and 18:00 in adolescent male volleyball players. Abbreviations: CAFcap, caffeine capsule; CAFrinse, caffeine mouth rinse; PLA, placebo; SJ, squat jump; CMJ, countermovement jump; PSQI, Pittsburgh Sleep Quality Index; rMEQ, reduced Morningness-Eveningness Questionnaire.

A randomized, double-blind, placebo-controlled crossover design was implemented to account for inter-individual variability and to eliminate potential order, learning, or circadian sequencing effects. Each participant completed nine experimental sessions corresponding to the factorial combination of three supplementation conditions administered via a double-dummy procedure: caffeine capsule (CAFcap), caffeine mouth rinse (CAFrinse), and placebo (PLA). In every session, each participant received both a capsule and a mouth-rinse solution; the active or inert nature of each formulation was determined by condition assignment, and three times of day (08:00, 12:00, 18:00). Both factors were counterbalanced across participants using a computer-generated 3 × 3 Latin-square procedure, in which each of the three supplementation conditions appeared exactly once across participant groups, controlling for condition-order carryover [27]. A standard 3 × 3 Latin square was applied exclusively to counterbalance the order of the three supplementation conditions (CAFcap, CAFrinse, PLA) across participants. This produced six possible condition sequences (3! = 6), to which the 24 participants were allocated in equal groups of four, requiring only a multiple of three rather than a complete factorial permutation. Time-of-day sessions (08:00, 12:00, 18:00) were administered in a fixed ascending order within each condition block, consistent with standard chronobiology crossover practice, which holds circadian phase constant to avoid cross-session sleep–wake disruption. Complete permutation of all possible condition-by-time-of-day sequences was neither intended nor necessary; the Latin square controlled exclusively for condition-order effects, which represent the primary source of systematic carryover bias in repeated-measures caffeine crossover designs. Allocation was concealed by an independent investigator who prepared the interventions and maintained code confidentiality until the completion of statistical analyses (Figure 1). A double-dummy procedure was employed so that, in every session, each participant received both a capsule (ingested with 250 mL of water, followed by a 30 min rest; plasma Tmax ≈ 45 min [10]) and a mouth-rinse solution (25 mL, swilled for 10–15 s and fully expectorated). In the CAFcap condition, an active caffeine capsule (3 mg·kg^−1^ anhydrous caffeine; Biotech USA, Budapest, Hungary) was paired with PLArinse; in the CAFrinse condition, PLAcap was paired with an active caffeine rinse (3 mg·kg^−1^ dissolved in 25 mL of water), with testing commencing immediately after expectoration to capture orosensory-mediated effects [18,19]. In the PLA condition, participants received both PLAcap and PLArinse. PLAcap consisted of an inert cellulose capsule identical in appearance and mass to the active capsule; PLArinse consisted of 25 mL of quinine-flavored water matched for volume, color, and bitterness to the active rinse. All formulations were indistinguishable to participants and to data-collection investigators, ensuring full blinding throughout.

Before the experimental phase, all athletes completed a familiarization session under the same environmental conditions (~22 °C; ~47% humidity). During this session, they practiced the entire testing battery, underwent anthropometric assessment for individualized dosing, and completed standardized questionnaires assessing habitual caffeine intake, sleep quality (PSQI ≤ 5; [28]), and circadian preference (rMEQ; [28]). Only players presenting good sleep quality and a “neither-type” chronotype were included. Participants were instructed to maintain regular sleep–wake behavior for 48 h before each trial, to abstain from caffeine, alcohol, and strenuous exercise for 24 h, and to consume their last meal at least 3 h before arrival. Morning sessions were scheduled at least 90 min after each athlete’s habitual wake time to minimize sleep inertia [29].

On each testing day, an independent researcher, who had no role in data collection, prepared and blinded the interventions. In the CAFcap condition, participants ingested anhydrous caffeine capsules (3 mg·kg^−1^; Biotech USA, Budapest, Hungary) with 250 mL of water, followed by a standardized 30 min absorption period (plasma Tmax ≈ 45 min). In the CAFrinse condition, they swilled an isodose caffeine solution (3 mg·kg^−1^ dissolved in 25 mL of water) for 10–15 s and fully expectorated it; testing began immediately thereafter to capture the orosensory-mediated effects documented in previous studies [18,19,30,31].

Each trial began with a 10 min seated stabilization period, followed by oral temperature measurement for at least 3 min using a calibrated digital thermometer (±0.05 °C accuracy). Participants then performed a standardized 10 min warm-up comprising 5 min of light jogging and dynamic mobility, 3 min of progressive accelerations, and 2 min of volleyball-specific movements such as approach runs and mock blocks.

All performance assessments were administered in the same fixed order across all trials to ensure reproducibility and minimize fatigue carry-over. The session began with lower-limb explosive tests: three squat jumps (SJ), each separated by 1 min of recovery, followed by a 2 min rest; then three countermovement jumps (CMJ), again separated by 1 min of rest, followed by another 2 min interval. This was followed by three block jumps, with 1 min between attempts and a 3 min recovery period thereafter to prevent neuromuscular interference with the subsequent agility assessment. Agility was evaluated using two trials of the 10 × 10 m *t*-test, separated by a 2 min passive recovery; a 5 min standardized rest interval ensured complete recovery before skill execution tests.

Volleyball-specific skills were assessed in the second part of the session. Players performed 10 spike attempts, each separated by approximately 10 s to preserve technical accuracy while avoiding fatigue accumulation. After a 3 min passive pause, athletes completed 10 serves using a self-toss technique, again with ~10 s of recovery between attempts.

Following each session, participants completed a structured questionnaire assessing acute caffeine-related side effects [32] comprising eight yes/no items covering increased urine output, gastrointestinal discomfort, tachycardia, headache, and related sensations. Blinding integrity was evaluated by asking each participant to identify the condition received (PLA, CAFcap, or CAFrinse) and to rate their confidence on a four-point scale (1 = guessing, 4 = certain), consistent with procedures reported in caffeine crossover trials [32]. Each trial was separated by 72 h to ensure full recovery and pharmacological washout, consistent with recommendations for crossover caffeine studies [33,34].

### 2.2. Participants

An a priori power analysis was performed using G*Power (version 3.1.9.7) for a repeated-measures ANOVA with a within-subject design (3 conditions × 3 times of day). The expected effect size (f = 0.25, corresponding to a medium effect under Cohen’s conventions) was anchored to countermovement jump height as the primary outcome variable, based on reported caffeine-induced improvements in vertical jump performance in trained athletes [6,26]. Given the developmental characteristics of the target population (16–18 years) and their low habitual caffeine intake (1.14 mg·kg^−1^·day^−1^), a conservative dose was selected. Recent investigations in adolescent athletes have shown that 3 mg·kg^−1^ of caffeine yields moderate improvements in cognitive and physical outcomes with minimal side effects. In contrast, higher doses disproportionately increase adverse events in low-consumer populations [13,19,26]. Using α = 0.05, statistical power (1 − β) = 0.80, and an estimated within-participant correlation of r = 0.50, the required sample size was calculated as *n* = 21 [28]. This calculation was based on expected main effects; statistical power for the Condition × Time-of-Day interaction terms, which constitute primary outcomes of this study, was not independently estimated and may have been insufficient to detect small interaction effect sizes. This constraint is acknowledged as a study limitation and does not affect the validity of the reported main-effect findings, for which the design was adequately powered. To account for potential attrition, 24 players were recruited. All randomized participants completed the protocol and were included in the final analyses. A total of 24 healthy male volleyball players (age: 16.9 ± 0.7 years; range: 16.0–18.0 years) met the eligibility criteria and participated in the study. Their mean stature, body mass, and body mass index were 177.0 ± 3.4 cm (range: 171.0–184.0 cm), 71.0 ± 4.2 kg (range: 64.0–80.0 kg), and 22.7 ± 1.7 kg·m^−2^ (range: 19.8–26.1 kg·m^−2^), respectively. Participants had accumulated 7.0 ± 1.0 years of structured volleyball training (range: 5.0–9.0 years). Inclusion criteria required ≥5 years of competitive experience, participation in ≥4 training sessions per week across the last 6 months, habitual caffeine intake < 3 mg·kg^−1^·day^−1^, good sleep quality (PSQI ≤ 5; [29], and an intermediate circadian preference on the reduced Morningness-Eveningness Questionnaire (rMEQ; five items) [30]. Exclusion criteria included smoking, alcohol consumption, chronic disease or medication use, recent injury (<3 months), stimulant or narcotic use, restrictive dieting, known caffeine allergy, or extreme circadian preference. Habitual caffeine intake was quantified using a validated 4-week semi-quantitative questionnaire [31], confirming that participants were mild caffeine consumers [32].

Written informed consent was obtained from all athletes and from parents/guardians for minors. The study protocol was approved by the University of Jendouba Ethics Committee (approval number: C-0018/2024, approval date: 18 November 2024) and conducted in accordance with the Declaration of Helsinki and its subsequent amendments.

### 2.3. Procedures

On each testing day, participants received one of the three supplementation conditions within the randomized, double-blind, placebo-controlled crossover design. In the caffeine capsule condition (CAFcap), athletes ingested anhydrous caffeine at a dose of 3 mg·kg^−1^ (BiotechUSA Kft., Budapest, Hungary) with 250 mL of water, followed by a standardized 30 min absorption period to coincide with the expected rise in plasma caffeine concentrations. In the caffeine mouth rinse condition (CAFrinse), the same absolute dose (3 mg·kg^−1^ dissolved in 25 mL of water) was swilled for 10–15 s and then completely expectorated; testing commenced immediately thereafter in accordance with protocols demonstrating rapid orosensory-driven effects on central nervous system activation [14,16,19].

#### 2.3.1. Jumping Assessment

After completing the warm-up, athletes performed the jump assessments, countermovement jump (CMJ), squat jump (SJ), attack jump, and block jump, in a standardized and fixed order across all sessions. For each test, three maximal attempts were executed, separated by 1 min of passive recovery, and the best performance was retained for analysis. All tests were performed on the same indoor wooden volleyball court, with athletes wearing their usual volleyball shoes to ensure ecological validity and consistent traction conditions. Jump height was measured using Optojump photoelectric cells (Microgate, Bolzano, Italy), an instrument shown to provide valid and reliable estimations of vertical jump performance [35].

For the CMJ, athletes started from an upright position with knees fully extended and hands placed on the hips to eliminate arm swing. Participants kept their feet shoulder-width apart and performed a self-selected countermovement before jumping as high as possible and landing under control [36]. The SJ was executed in the same stance, with hands on hips; athletes descended into a half-squat position and maintained this posture for approximately 3 s to eliminate any contribution from the stretch–shortening cycle before performing a purely concentric vertical jump [37].

The block-and-attack jumps followed standardized volleyball-specific movement patterns. For the block jump, players started from a stationary position with hands at chest height; they performed a rapid countermovement followed by an explosive extension, reaching upward with fully extended arms at takeoff. Squat depth and arm motion were kept consistent with players’ habitual blocking technique [38]. The attack jump replicated each athlete’s natural spiking approach. The approach distance (typically 2–3 steps) was standardized individually during familiarization, and athletes used their habitual arm swing to perform a maximal jump, allowing the assessment of game-relevant jump performance.

To ensure maximal validity and prevent fatigue contamination between tests, standardized verbal encouragement was provided during every trial, while no feedback on jump height was given. All players were familiarized with the procedures in a separate session, and no learning effects were observed during the study.

#### 2.3.2. Change-of-Direction Speed (CODS) Assessment

**Figure 2:**
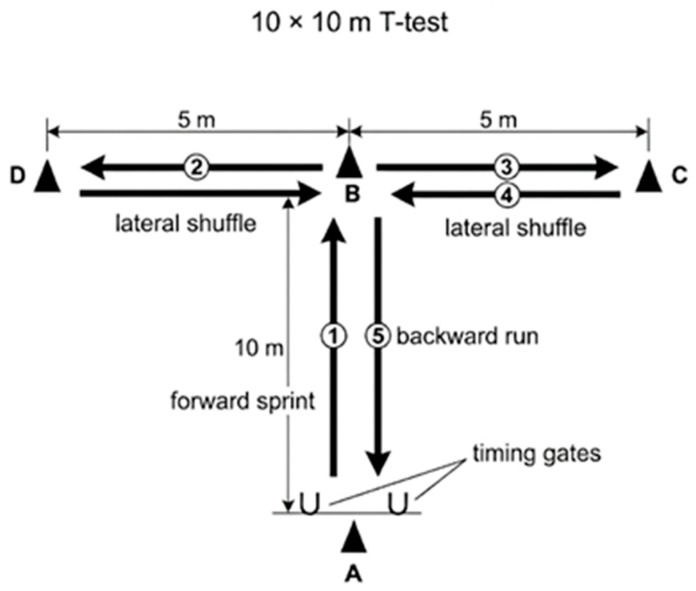
Schematic representation of the 10 × 10 m *t*-test used to assess change-of-direction speed. Athletes started at point A, with the toes positioned 0.3 m behind the first timing gate, sprinted forward to point B, shuffled laterally to points D and C, returned to point B, and then ran backward to the start/finish line. During lateral movements, participants were instructed not to cross their feet. The fastest of two maximal trials was retained for analysis.

Change-of-direction speed was evaluated using the standardized *t*-test, following the procedures described by Miller et al. [39], and adapted to a 10 × 10 m configuration in line with international recommendations. All assessments were conducted on the same indoor volleyball court as the other performance tests to ensure uniform surface and traction conditions. The course was marked precisely according to the reference layout (Figure 2). Performance times were recorded using a photocell timing system (Witty, Microgate, Bolzano, Italy), previously validated for its high reliability in agility and CODS measurements.

Each trial began from a standing start with the athlete’s toes positioned 0.3 m behind the first timing gate to prevent premature triggering of the sensors. Upon the investigator’s verbal signal, participants sprinted forward at maximal effort before executing the prescribed sequence of lateral shuffles and backward running. During lateral movements, athletes were instructed to lead with their preferred leg and to avoid crossing their feet, ensuring consistency with natural volleyball-specific movement patterns and enhancing ecological validity [40].

Two maximal trials were performed, with 2 min of passive recovery between attempts to limit fatigue and maintain test reproducibility. Standardized verbal encouragement was provided, while performance feedback was withheld to prevent pacing bias. The fastest trial was retained for subsequent analyses.

#### 2.3.3. Volleyball Specific-Skills Assessment

**Figure 3:**
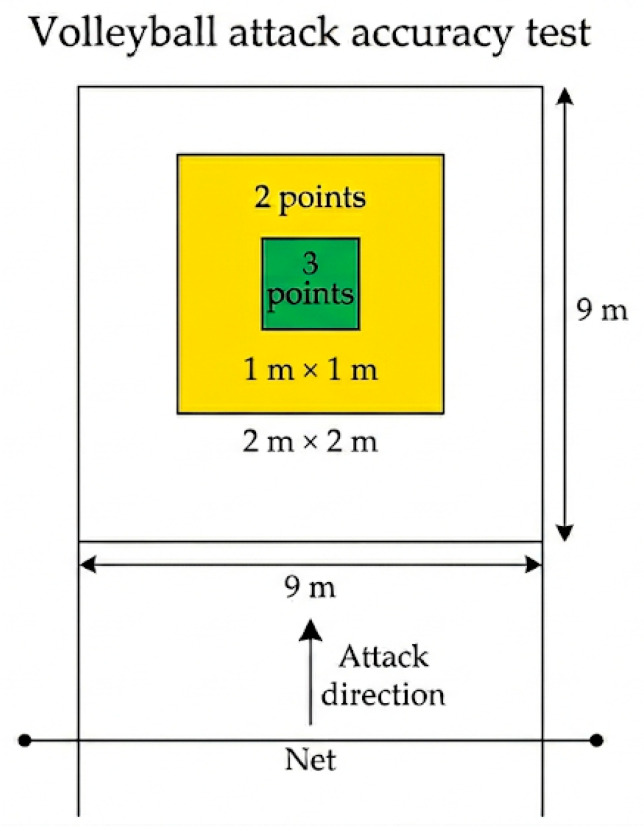
Schematic representation of the volleyball attack accuracy test. The target half-court (9 m × 9 m) was divided into predefined scoring zones, with 3 points awarded for attacks landing in the central green zone (1 m × 1 m) and 2 points for attacks landing in the surrounding yellow zone (2 m × 2 m). All other in-bounds attacks were scored 1 point, whereas balls landing into the net or out of bounds received 0 points.

Volleyball-specific skills were evaluated through standardized tests of spike and serve accuracy, conducted on an official indoor court (9 m × 18 m) with a fixed net height of 2.43 m. Attack accuracy was assessed using a modified and validated protocol designed for junior volleyball players [1]. The attack target area was divided into predefined scoring zones (Figure 3), with 3 points awarded for successful hits in the central green zone (1 m × 1 m), 2 points for hits in the surrounding yellow zone (2 m × 2 m), and 1 point for successful attacks landing elsewhere within the court boundaries. Any ball landing out of bounds or into the net was assigned a score of 0. To ensure consistency across trials, the same experienced setter delivered all sets, and players executed each attack using their habitual approach and arm swing from their regulation playing position.

**Figure 4:**
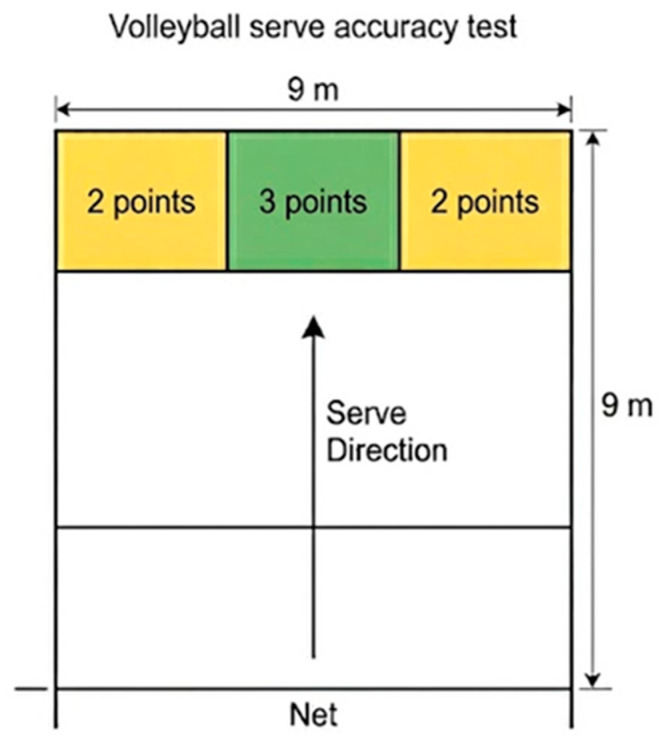
Schematic representation of the volleyball serve accuracy test. The target half-court (9 m × 9 m) was divided into predefined scoring zones: the central green zone was worth 3 points, and the adjacent yellow zones were worth 2 points. All other in-bounds areas were scored 1 point, whereas serves landing into the net or out of bounds received 0 points.

Service accuracy was evaluated using the protocol proposed by [41], adapted for adolescent volleyball athletes. The scoring system paralleled that of the attack test but used zones of slightly different dimensions (Figure 4): 3 points for the central green zone (1 m × 0.75 m) and 2 points for the yellow zone (1 m × 2 m). Players performed each serve using their natural self-toss technique and serving rhythm. As with the attack test, out-of-bounds or net-contact serves were scored 0.

For both skills assessments, players completed 10 attempts, and the cumulative score was used for performance analysis. Two independent observers recorded all outcomes, and scoring agreement between observers was complete. All tests were performed using a standardized Molten V5M5000 volleyball, identical to the ball used in regular training and competition (Figure 3 and Figure 4).

#### 2.3.4. Side Effects

Immediately after each testing session, participants completed a standardized questionnaire assessing potential caffeine-related side effects, including increased urine output, gastrointestinal discomfort, tachycardia, headache, or any other unusual sensations. The questionnaire consisted of eight yes/no items adapted from the instrument developed by [32], which has been widely used to monitor acute caffeine tolerability in sports science research.

### 2.4. Statistical Analysis

Before model fitting, distributional assumptions were evaluated for each of the seven dependent variables (SJ, CMJ, block jump, attack jump, CODS, spike accuracy, serve accuracy): normality of residuals was assessed using the Shapiro–Wilk test for each of the seven dependent variables across all nine within-subject cells (three conditions × three times of day); all outcomes satisfied the normality assumption (all *p* > 0.05), and no data transformation was required. Sphericity was examined using Mauchly’s test; Greenhouse–Geisser corrections were applied where sphericity was violated. Each outcome was then analyzed using a two-way repeated-measures ANOVA with two within-subject factors: Condition (PLA, CAFcap, CAFrinse; 3 levels) and Time of Day (08:00, 12:00, 18:00; 3 levels), yielding a 3 × 3 within-subject factorial structure (nine cell means per outcome). When a significant main effect or interaction was observed, Bonferroni-adjusted pairwise comparisons were performed, as appropriate, to examine differences between supplementation conditions within each time point and/or differences between time points within each condition. Statistical significance was set at *p* ≤ 0.05 (two-tailed). The seven dependent variables (SJ, CMJ, block jump, attack jump, CODS, spike accuracy, serve accuracy) were analyzed in separate two-way repeated-measures ANOVA models, as they represent conceptually and physiologically distinct performance constructs. No cross-test omnibus correction was applied; however, the resulting inflation of the family-wise Type I error rate across seven tests is acknowledged as a limitation, and readers should interpret borderline significant findings with appropriate caution. Effect sizes for omnibus ANOVA effects were reported as partial eta squared (ηp^2^), with thresholds of approximately 0.01, 0.06, and 0.14 representing small, medium, and large effects, respectively. Effect sizes for within-subject pairwise comparisons were calculated as Cohen’s d, defined as the mean paired difference divided by the standard deviation of the paired differences, with values of approximately 0.20, 0.50, and 0.80 interpreted as small, medium, and large, respectively. For categorical outcomes (adverse events, yes/no), we analyzed them separately for each time of day using Cochran’s Q test across the three paired conditions (PLA, CAFcap, and CAFrinse). When the overall Cochran’s Q test was significant, post hoc pairwise comparisons were conducted using McNemar tests with continuity correction, and exact *p* values were reported when discordant counts were small. Holm adjustment was applied to account for multiple pairwise comparisons. Adverse-event data are reported as n (%), together with the number of discordant pairs per McNemar test. All analyses were performed using SPSS (version 28; IBM Corp., Armonk, NY, USA), and the significance threshold was set at alpha = 0.05 for all tests.

## 3. Results

**Figure 5:**
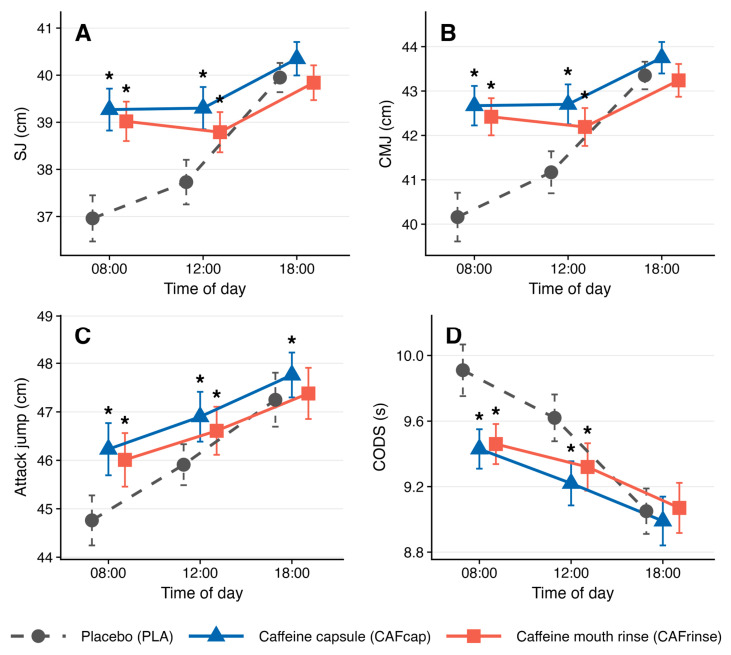
Estimated marginal means (±1 standard error of the mean, SE) for squat jump height (**A**), countermovement jump height (**B**), attack jump height (**C**), and change-of-direction speed ((**D**); 10 × 10 m *t*-test) under placebo (PLA), caffeine capsule (CAFcap; 3 mg·kg^−1^), and caffeine mouth rinse (CAFrinse; 3 mg·kg^−1^) at 08:00, 12:00, and 18:00 in trained adolescent male volleyball players (*n* = 24). These four outcomes exhibited significant Condition × Time-of-Day interaction effects (two-way repeated-measures ANOVA; *p* ≤ 0.005; see Table 2). Asterisks (*) denote significant differences relative to PLA within the corresponding time point (*p* < 0.05, Bonferroni-adjusted pairwise comparisons). The convergence of CAFcap and CAFrinse trajectories toward the PLA trajectory at 18:00 reflects the attenuation of caffeine ergogenicity as neuromuscular performance approaches its natural circadian peak. For CODS (**D**), lower values indicate faster performance.

All seven outcomes demonstrated significant main effects of both Condition and Time of Day, with all effect sizes classified as large (ηp^2^ range: 0.345–0.891; Table 1). Significant Condition × Time-of-Day interactions were observed for squat jump (SJ; F(4, 92) = 21.91, *p* < 0.001, ηp^2^ = 0.488), countermovement jump (CMJ; F(4, 92) = 19.29, *p* < 0.001, ηp^2^ = 0.456), attack jump (F(4, 92) = 4.06, *p* = 0.005, ηp^2^ = 0.150), and change-of-direction speed (CODS; F(4, 92) = 10.75, *p* < 0.001, ηp^2^ = 0.318), indicating time-dependent modulation of caffeine ergogenicity. Interactions were non-significant for block jump (F(4, 92) = 0.48, *p* = 0.750, ηp^2^ = 0.020), spike accuracy (F(4, 92) = 1.22, *p* = 0.308, ηp^2^ = 0.050), and serve accuracy (F(4, 92) = 0.84, *p* = 0.504, ηp^2^ = 0.035) (Figure 5). Descriptive statistics and Bonferroni-adjusted pairwise comparisons for all conditions and time points are reported in Table 2.

### 3.1. Outcomes with Significant Condition × Time-of-Day Interactions

For SJ, CMJ, and attack jump, ergogenic effects were most pronounced at 08:00 and 12:00 and were attenuated or absent at 18:00 as performance approached its natural diurnal peak. For CODS, a parallel pattern was observed, with benefits fully abolished by 18:00.

At 08:00, both CAFcap and CAFrinse yielded significantly greater SJ heights than PLA (CAFcap: +2.31 cm, 95% CI [1.88, 2.74], dz = 2.26; CAFrinse: +2.06 cm, 95% CI [1.62, 2.50], dz = 1.99) and CMJ heights CAFcap: +2.51 cm, 95% CI [1.80, 3.22], dz = 1.49; CAFrinse: +2.26 cm, 95% CI [1.54, 2.98], dz = 1.33, with no significant inter-condition difference for either outcome (both dz ≤ 0.47). At 12:00, significant benefits persisted under both caffeine conditions for SJ and CMJ relative to PLA (dz range: 0.97–1.91); CAFcap exceeded CAFrinse exclusively for SJ (+0.51 cm, 95% CI [0.14, 0.88], dz = 0.57), representing the sole significant inter-condition difference at this time point. At 18:00, neither caffeine condition differed significantly from PLA for SJ or CMJ, although CAFcap remained marginally higher than CAFrinse for both outcomes (dz = 0.65). SJ and CMJ improved significantly from 08:00 to 18:00 under PLA (dz = 1.70 and 1.42, respectively) and under both caffeine conditions (dz range: 0.56–0.98), confirming an independent diurnal effect; the compressed diurnal amplitude under caffeine relative to PLA indicates partial attenuation of circadian performance variation.

For attack jump, significant improvements over PLA were observed at 08:00 (CAFcap: +1.47 cm, 95% CI [1.04, 1.90], dz = 1.41; CAFrinse: +1.25 cm, 95% CI [0.80, 1.70], dz = 1.14) and 12:00 (CAFcap: dz = 0.99; CAFrinse: dz = 0.61), with no significant inter-condition difference at either time point (dz ≤ 0.37). At 18:00, only CAFcap retained a significant advantage over PLA (+0.51 cm, 95% CI [0.11, 0.91], dz = 0.53), whereas CAFrinse did not differ significantly from PLA (dz = 0.13).

For CODS, both CAFcap (−0.48 s, 95% CI [−0.60, −0.36], dz = 1.70) and CAFrinse (−0.45 s, 95% CI [−0.57, −0.33], dz = 1.58) significantly reduced *t*-test time relative to PLA at 08:00, with no significant inter-condition difference (dz = 0.24). Benefits were attenuated at 12:00 (CAFcap: dz = 1.11; CAFrinse: dz = 0.89) and entirely absent at 18:00, by which point PLA performance had improved to 9.05 ± 0.68 s, eliminating any ergogenic margin. *t*-test performance improved significantly from 08:00 to 18:00 under all conditions (PLA: dz = 1.17; CAFcap: dz = 0.63; CAFrinse: dz = 0.64).

### 3.2. Outcomes Without a Significant Condition × Time-of-Day Interaction

Block jump, spike accuracy, and serve accuracy exhibited consistent caffeine-related improvements across all three time points, irrespective of circadian phase. For block jump, both CAFcap and CAFrinse improved performance relative to PLA at 08:00 (+1.02 cm, 95% CI [0.58, 1.46], dz = 0.96 and +0.92 cm, 95% CI [0.52, 1.32], dz = 0.95, respectively), 12:00 (dz = 0.73 and 0.69, respectively), and 18:00 (CAFcap: +1.19 cm, 95% CI [0.74, 1.64], dz = 1.10; CAFrinse: +0.87 cm, 95% CI [0.35, 1.39], dz = 0.69), with no meaningful inter-condition differences at any time point (all dz ≤ 0.30). A progressive diurnal improvement was observed under all three conditions (PLA, 08:00 to 18:00: dz = 1.75; Table 2).

For spike accuracy, both caffeine conditions improved scores relative to PLA at all time points (dz range: 0.71–1.35). At 08:00, CAFcap yielded +1.04 points (95% CI [0.64, 1.44], dz = 1.09) and CAFrinse yielded +1.54 points (95% CI [1.06, 2.02], dz = 1.35) over PLA; the inter-condition difference was non-significant (dz = 0.36). At 12:00, significant improvements over PLA were maintained (CAFcap: +0.70 points, 95% CI [0.28, 1.12], dz = 0.71; CAFrinse: +1.08 points, 95% CI [0.65, 1.51], dz = 1.06; inter-condition difference non-significant, dz = 0.36). At 18:00, improvements over PLA were equivalent across both caffeine conditions (both +1.12 points, 95% CI [0.63, 1.61], dz = 0.97). Spike accuracy increased progressively from 08:00 to 18:00 under all conditions, including PLA (dz = 1.85), confirming a diurnal rhythm in technical skill execution independent of supplementation.

For serve accuracy, significant improvements over PLA were confirmed for CAFcap at 08:00 (+0.96 points, 95% CI [0.23, 1.69], dz = 0.54) and 18:00 (+1.66 points, [1.03, 2.29], dz = 1.07), and for CAFrinse at 08:00 (+1.08 points, 95% CI [0.33, 1.83], dz = 0.59), 12:00 (+1.09 points, 95% CI [0.47, 1.71], dz = 0.72), and 18:00 (+1.04 points, 95% CI [0.44, 1.64], dz = 0.71). The CAFcap versus PLA comparison at 12:00 did not survive Bonferroni adjustment (dz = 0.52). No significant inter-condition differences were observed at any time point (all dz ≤ 0.36). Serve accuracy was significantly higher at 18:00 than at 08:00 under all conditions (PLA: dz = 1.00; CAFcap: dz = 1.40; CAFrinse: dz = 0.81), consistent with the independent diurnal effect observed across the non-interaction outcome group.

### 3.3. Side Effects

Adverse events. Adverse events were infrequent overall and were analyzed separately within each time of day across the three paired conditions (PLA, CAFcap, and CAFrinse). At 08:00, adverse events were reported in 4.2%, 16.7%, and 0% of participants in the PLA, CAFcap, and CAFrinse groups, respectively, with a significant overall effect (Cochran’s Q = 6.50, *p* = 0.039). Nevertheless, no pairwise McNemar comparison remained significant after Holm correction. At 12:00, adverse events were reported in 0%, 25.0%, and 4.2% of participants under PLA, CAFcap, and CAFrinse, respectively, again yielding a significant overall effect (Cochran’s Q = 8.86, *p* = 0.012), although none of the post hoc pairwise comparisons survived Holm adjustment. At 18:00, no adverse events were reported in any condition. Collectively, these findings suggest that adverse events were uncommon, were observed primarily after CAFcap, and were not reported during evening testing.

## 4. Discussion

The present study examined the acute effects of caffeine, administered as a capsule or a mouth rinse, on lower-limb power and volleyball-specific technical performance across three times of day in trained adolescent male volleyball players. The principal findings were fourfold. First, both caffeine conditions improved several physical and technical outcomes relative to placebo, confirming the general ergogenic potential of caffeine for short-duration, high-intensity, and skill-based tasks [6,42]. Second, the benefits for SJ, CMJ, and attack jump were most evident at 08:00 and 12:00, suggesting that caffeine partly attenuated the early-day decrement in explosive performance. Third, caffeine capsule ingestion showed only a modest and outcome-specific advantage over mouth rinsing, mainly for some power variables at midday, whereas both delivery modes were broadly comparable for volleyball-specific accuracy. Fourth, irrespective of supplementation, most outcomes improved progressively from morning to evening, consistent with the expected diurnal increase in neuromuscular and technical readiness [22,23,24]. These findings partially support our first hypothesis: CAFcap showed a statistically significant advantage over CAFrinse for a limited number of lower-limb power outcomes at midday, but inter-condition differences were absent for CODS, block jump, spike accuracy, and serve accuracy at all time points, indicating that capsule superiority is neither consistent nor large in magnitude across the full performance battery.

The restriction of enrolment to individuals with intermediate circadian preferences was intended to minimize the confounding influence of circadian misalignment on baseline performance, thereby isolating the effects of supplementation and time of day [25]. The larger ergogenic effects observed in the morning and, to a lesser extent, at midday for SJ, CMJ, and attack jump align with previous evidence showing that caffeine can attenuate time-of-day-related declines in cognitive and short-term maximal performance in youth athletes [13,43,44]. Mechanistically, this pattern is plausible because caffeine acts primarily as an adenosine receptor antagonist, thereby increasing arousal, reducing perceived fatigue, and facilitating central motor drive [10]. Such effects may enhance motor unit recruitment and the rate of force development, which are critical for explosive tasks such as SJ and CMJ [37,45]. In parallel, chronobiological literature has consistently shown that muscular power and psychomotor performance are often reduced earlier in the day and tend to peak in the late afternoon or early evening, likely due to combined influences of sleep inertia, body temperature, endocrine rhythms, and neuromuscular efficiency [22,23,25,46]. Our data fit this framework closely: when baseline performance was relatively low at 08:00 and 12:00, caffeine provided meaningful support; when performance approached its natural peak at 18:00, the additional effect was attenuated or absent.

Comparing caffeine delivery modes provides additional practical and mechanistic insights. Although capsule ingestion tended to produce slightly greater benefits for some lower-limb power outcomes, particularly at 12:00, caffeine mouth rinsing was also clearly effective overall. This pattern suggests that full systemic absorption may not be essential to elicit meaningful ergogenic effects in this population and setting. Caffeine mouth rinsing has been proposed to stimulate oral bitter taste receptors and afferent pathways projecting to cortical and subcortical regions involved in arousal, attention, and motor preparation, thereby facilitating performance even in the absence of ingestion [14,15]. Consistent with established protocols [18], the rinse volume in this study was 25 mL, yielding a concentrated solution with marked bitterness that could theoretically serve as an independent sensory signal. While this concentration ensured full dose delivery within a brief oral exposure window, the bitterness intensity may have helped participants identify the active rinse condition, a factor that partly limits blinding integrity and should be considered when interpreting mouth-rinse efficacy in ecologically sensitive settings [18,31]. This view is reinforced by previous findings showing that caffeine mouth rinsing can improve strength, muscular endurance, and cognitive performance, indicating that oral sensory exposure alone may produce meaningful central effects even in the absence of conventional ingestion [20]. Additional support comes from studies reporting protocol-dependent, yet potentially meaningful, effects of caffeine rinsing on physical and cognitive performance, particularly during brief tasks that rely heavily on central activation [18,31]. At the same time, the modest advantage observed with capsule ingestion for some jump outcomes remains biologically plausible, as systemic caffeine availability may provide a stronger or more sustained stimulus to neuromuscular output than an exclusively orosensory intervention [10]. Accordingly, the present findings suggest that capsule ingestion and mouth rinsing represent context-dependent alternative strategies, each with distinct practical applications, to be selected according to the specific demands and constraints of the setting rather than used concurrently.

The effects observed for spike and serve accuracy are particularly relevant from a volleyball-specific perspective. Volleyball performance depends not only on muscular power, but also on visual tracking, anticipatory timing, sensorimotor coordination, and the capacity to reproduce precise motor patterns under time pressure [1,41]. The improvement in technical accuracy under both caffeine conditions suggests that caffeine may have enhanced psychomotor function beyond its effects on force production. This interpretation is compatible with the broader literature showing that caffeine can improve vigilance, reaction speed, and motor execution, especially when alertness is suboptimal [10,11]. It is also noteworthy that the two delivery modes produced broadly similar effects on spike and serve accuracy. For technically demanding actions, central arousal and response readiness may be especially important, which may help explain why an orosensory stimulus such as mouth rinsing can approach the effectiveness of capsule ingestion. Moreover, the progressive increase in technical performance from morning to evening across conditions suggests that circadian phase exerts an independent influence on skill execution, likely through daily variation in alertness, perceptual processing, and neuromuscular coordination [22,23]. In this sense, caffeine appears to complement, rather than replace, the natural rhythm of technical readiness.

An additional practical finding concerns tolerability. Although adverse events were infrequent and no serious adverse event was recorded, mild complaints occurred more often after caffeine capsule ingestion than after caffeine mouth rinsing, particularly at 08:00 and 12:00, whereas none were reported at 18:00. Headache was the most frequent complaint, which is consistent with the greater systemic exposure associated with capsule ingestion in low-habitual caffeine consumers. These findings suggest that caffeine mouth rinsing may offer a useful compromise for adolescent athletes, providing meaningful ergogenic benefits with fewer adverse events. Nevertheless, this interpretation should remain cautious because the number of events was low, symptom severity was not graded, and the study was not powered specifically to detect between-condition differences in tolerability.

From an applied standpoint, these findings have several implications for coaches, strength-and-conditioning staff, and sport federations working with adolescent volleyball players. First, when training sessions or competitive matches are scheduled early in the morning, a low-dose caffeine strategy may help offset the disadvantage of reduced early-day neuromuscular readiness. Second, caffeine mouth rinsing is a practical alternative when rapid administration is needed, or gastrointestinal tolerance is a concern; however, its use in indoor volleyball competition settings requires consideration of strict floor hygiene regulations that may preclude expectoration on or near the court, limiting its real-match applicability. Third, capsule ingestion may still be preferable when maximizing lower-limb power is the primary objective and when sufficient time is available for absorption. Fourth, the more limited additional benefit observed at 18:00 suggests that caffeine use later in the day should be individualized rather than systematic, especially in adolescents, for whom unnecessary evening stimulation could be undesirable. In practical terms, these data support a context-dependent strategy: caffeine may be most valuable when baseline readiness is compromised by time of day, and mouth rinsing may be particularly useful when logistical simplicity and tolerability are priorities [10,14]. From a regulatory standpoint, the 3 mg·kg^−1^ dose used in this study falls within currently permitted limits under the WADA framework, and no urinary threshold has been re-established since 2004 [9,10]. Nonetheless, practitioners working with adolescent athletes should verify compliance with federation-specific regulations and ensure that athletes receive appropriate education on responsible caffeine use.

Several limitations should be acknowledged. First, the study involved only trained adolescent male volleyball players with low habitual caffeine intake and a restricted chronotype profile, which limits generalizability to female athletes, adults, highly habituated caffeine users, or athletes with more extreme circadian preferences. Second, although the selected performance tests were ecologically relevant, the design remained experimental rather than truly competitive, and transfer to real match situations should therefore be interpreted cautiously. Third, the a priori power calculation was based on the main effect of condition; statistical power for the Condition × Time-of-Day interaction terms, which represent the primary outcomes of this study, was not independently estimated and may have been insufficient to reliably detect small interaction effect sizes. Fourth, plasma or salivary caffeine concentrations were not measured, so the pharmacokinetic contribution of capsule ingestion and the extent of any systemic exposure after rinsing cannot be directly confirmed. Fifth, no mechanistic biomarkers, such as electromyography, hormonal responses, or continuous core temperature, were collected, which limits causal inference into the pathways responsible for the observed performance changes. Finally, despite the double-blind design, blinding integrity may have been compromised in the capsule condition by the distinct arousal profile of systemic caffeine and in the rinse condition by the bitterness intensity relative to the trace-quinine placebo, both of which could permit condition identification. Although the study was designed as a double-blind study, conclusive blinding data were not available for formal analysis. Therefore, expectancy effects and placebo responses cannot be fully disentangled from pharmacological mechanisms, and this should be considered when interpreting condition-specific effect sizes. Future studies should integrate pharmacokinetic and mechanistic measurements, include female and mixed-sex youth cohorts, test different doses and rinse durations, and examine these strategies in more ecologically relevant volleyball contexts, such as match simulations and congested tournament formats.

## 5. Conclusions

In conclusion, these data suggest that both caffeine capsule ingestion and caffeine mouth rinsing can enhance lower-limb power and volleyball-specific technical accuracy in trained adolescent male players, with the greatest benefits occurring earlier in the day, when baseline performance is lower. Capsule ingestion may retain a small advantage for vertical jump height and change-of-direction speed under certain time-of-day conditions, whereas mouth rinsing appears to be a credible, practical alternative for youth volleyball settings when logistical or tolerability constraints preclude ingestion.

## Figures and Tables

**Table 1:** Two-way repeated-measures ANOVA omnibus effects (Panel A) and Bonferroni-adjusted pairwise comparisons between active conditions and placebo (Panel B) for all performance outcomes (*n* = 24).

**Panel A—Omnibus ANOVA Effects**									
**Outcome**	**Condition F(2, 46)**	***p***	**ηp^2^**	**Time of Day F(2, 46)**	***p***	**ηp^2^**	**Condition × TOD F(4, 92)**	***p***	**ηp^2^**
SJ	48	<0.001	0.676	38.59	<0.001	0.627	21.91	<0.001	0.488
CMJ	38.76	<0.001	0.628	33.5	<0.001	0.593	19.29	<0.001	0.456
Block jump	31.65	<0.001	0.579	132.54	<0.001	0.852	0.48	0.75	0.02
Attack jump	44.4	<0.001	0.659	105.73	<0.001	0.821	4.06	0.005	0.15
CODS	29.61	<0.001	0.563	12.1	<0.001	0.345	10.75	<0.001	0.318
Spike accuracy	48.7	<0.001	0.679	187.6	<0.001	0.891	1.22	0.308	0.05
Serve accuracy	21.67	<0.001	0.485	28.95	<0.001	0.557	0.84	0.504	0.035
**Panel B—Pairwise Comparisons: Active Conditions vs. Placebo and Between Active Conditions**									
**Outcome**	**TOD**	**CAFcap vs. PLA MD**	**95% CI**	**dz**	**CAFrinse vs. PLA MD**	**95% CI**	**dz**	**CAFcap vs. CAFrinse dz**	
**SJ (cm)**	8:00	2.31	[1.88, 2.74]	2.26	2.06	[1.62, 2.50]	1.99	0.47	
	12:00	1.57	[1.19, 1.95]	1.76	1.06	[0.60, 1.52]	0.97	0.57 †	
	18:00	—	—	ns	—	—	ns	0.65 †	
**CMJ (cm)**	8:00	2.51	[1.80, 3.22]	1.49	2.26	[1.54, 2.98]	1.33	0.47	
	12:00	1.53	[1.19, 1.87]	1.91	1.02	[0.58, 1.46]	0.98	0.57 †	
	18:00	—	—	ns	—	—	ns	0.65 †	
**Block jump (cm)**	8:00	1.02	[0.57, 1.47]	0.96	0.92	[0.51, 1.33]	0.95	0.09	
	12:00	0.8	[0.34, 1.26]	0.73	0.76	[0.30, 1.22]	0.69	0.03	
	18:00	1.19	[0.73, 1.65]	1.1	0.87	[0.34, 1.40]	0.69	0.3	
**Attack jump (cm)**	8:00	1.47	[1.03, 1.91]	1.41	1.25	[0.79, 1.71]	1.14	0.19	
	12:00	0.99	[0.57, 1.41]	0.99	0.7	[0.22, 1.18]	0.61	0.37	
	18:00	0.51	[0.10, 0.92]	0.53	—	—	0.13	0.42	
**CODS (s)**	8:00	−0.48	[−0.60, −0.36]	1.7	−0.45	[−0.57, −0.33]	1.58	0.24	
	12:00	−0.40	[−0.55, −0.25]	1.11	−0.30	[−0.44, −0.16]	0.89	0.29	
	18:00	—	—	ns	—	—	ns	—	
**Spike accuracy (pts)**	8:00	1.04	[0.64, 1.44]	1.09	1.54	[1.06, 2.02]	1.35	0.38	
	12:00	0.7	[0.28, 1.12]	0.71	1.08	[0.65, 1.51]	1.06	0.37	
	18:00	1.12	[0.63, 1.61]	0.97	1.12	[0.63, 1.61]	0.97	0	
**Serve accuracy (pts)**	8:00	0.96	[0.21, 1.71]	0.54	1.08	[0.31, 1.85]	0.59	0.07	
	12:00	— (ns) ‡	—	0.52	1.09	[0.45, 1.73]	0.72	0.04	
	18:00	1.66	[1.01, 2.31]	1.07	1.04	[0.42, 1.66]	0.71	0.36	

TOD = time of day; SJ = squat jump; CMJ = countermovement jump; CODS = change-of-direction speed (10 × 10 m *t*-test); pts = cumulative accuracy score; MD = mean difference (active minus PLA; for CODS, negative values indicate faster performance); 95% CI = 95% confidence interval for MD; dz = Cohen’s dz (paired within-subject effect size); — = comparison not statistically significant (*p* > 0.05, Bonferroni-adjusted) and MD/CI therefore not reported; ns = non-significant. † Significant difference between CAFcap and CAFrinse (*p* < 0.05, Bonferroni-adjusted). ‡ CAFcap vs. PLA at 12:00 for serve accuracy: the raw comparison yielded dz = 0.52 but did not survive Bonferroni adjustment (*p* > 0.05). **Panel A:** Greenhouse-Geisser corrections were applied where Mauchly’s test indicated a violation of sphericity. Effect size thresholds: ηp^2^ = 0.01 small, 0.06 medium, 0.14 large. All significant effects (*p* < 0.05) are presented; all F-ratios are reported to two decimal places. **Panel B:** Mean differences are computed as the arithmetic difference between condition means reported in Table 2. Ninety-five percent CIs are derived from reported MD and Cohen’s dz values using the formula CI = MD ± t(23, 0.025) × (MD/dz)/√24, where t(23, 0.025) = 2.069. For CAFcap vs. CAFrinse, only dz is reported, as MD and CI were not reported in the source analysis. Cohen’s dz thresholds: 0.20 small, 0.50 medium, 0.80 large.

**Table 2:** Performance outcomes (mean ± SD) under placebo (PLA), caffeine capsule (CAFcap), and caffeine mouth rinse (CAFrinse) at 08:00, 12:00, and 18:00 in trained adolescent male volleyball players (*n* = 24), with Bonferroni-adjusted pairwise significance notation and within-condition diurnal effect sizes (Cohen’s dz, 08:00 to 18:00).

**Outcome**	**08:00 PLA**	**08:00 CAFcap**	**08:00 CAFrinse**	**12:00 PLA**	**12:00 CAFcap**	**12:00 CAFrinse**	**18:00 PLA**	**18:00 CAFcap**	**18:00 CAFrinse**
SJ (cm)	36.96 ± 2.40	39.27 ± 2.18 *	39.02 ± 2.05 *	37.73 ± 2.32	39.30 ± 2.20 *†	38.79 ± 2.09 *	39.95 ± 1.52 ^a^	40.35 ± 1.74 †^a^	39.84 ± 1.81 ^ab^
CMJ (cm)	40.16 ± 2.69	42.67 ± 2.18 *	42.42 ± 2.05 *	41.17 ± 2.32 ^a^	42.70 ± 2.20 *†	42.19 ± 2.09 *	43.35 ± 1.52 ^ab^	43.75 ± 1.74 †^ab^	43.24 ± 1.81 ^ab^
Block jump (cm)	41.14 ± 2.68	42.16 ± 2.64 *	42.06 ± 2.85 *	42.18 ± 2.72 ^a^	42.98 ± 2.61 *^a^	42.94 ± 2.50 *^a^	42.95 ± 2.54 ^ab^	44.14 ± 2.47 *^ab^	43.82 ± 2.21 *^ab^
Attack jump (cm)	44.76 ± 2.53	46.23 ± 2.64 *	46.01 ± 2.71 *	45.91 ± 2.07 ^a^	46.90 ± 2.52 *^a^	46.61 ± 2.43 *^a^	47.25 ± 2.72 ^ab^	47.76 ± 2.26 *^ab^	47.38 ± 2.58 ^ab^
CODS (s)	9.91 ± 0.77	9.43 ± 0.59 *	9.46 ± 0.60 *	9.62 ± 0.70 ^a^	9.22 ± 0.66 *^a^	9.32 ± 0.71 *^a^	9.05 ± 0.68 ^ab^	8.99 ± 0.73 ^a^	9.07 ± 0.75 ^a^
Spike accuracy (pts)	14.92 ± 3.11	15.96 ± 2.94 *	16.46 ± 2.81 *	16.13 ± 3.29 ^a^	16.83 ± 3.17 *^a^	17.21 ± 3.16 *^a^	17.42 ± 3.23 ^ab^	18.54 ± 2.84 *^ab^	18.54 ± 3.05 *^ab^
Serve accuracy (pts)	16.54 ± 1.82	17.50 ± 1.32 *	17.62 ± 2.06 *	17.12 ± 2.09	18.12 ± 1.98	18.21 ± 2.13 *	17.96 ± 1.68 ^a^	19.62 ± 1.69 *^ab^	19.00 ± 1.93 *^a^
**Diurnal dz (08:00→18:00)**									
SJ	—	—	—	—	—	—	1.7	0.98	0.56
CMJ	—	—	—	—	—	—	1.42	0.98	0.56
Block jump	—	—	—	—	—	—	1.75	1.93	1.62
Attack jump	—	—	—	—	—	—	2.71	1.34	1.25
CODS	—	—	—	—	—	—	1.17	0.63	0.64
Spike accuracy	—	—	—	—	—	—	1.85	2.78	1.67
Serve accuracy	—	—	—	—	—	—	1	1.4	0.81

SJ = squat jump; CMJ = countermovement jump; CODS = change-of-direction speed (10 × 10 m *t*-test; lower values indicate faster performance); pts = cumulative accuracy score (maximum 30 points per 10 attempts). All diurnal dz values (08:00 to 18:00) were statistically significant (*p* < 0.05) after Bonferroni adjustment within each condition. * Significant difference relative to PLA within the same time point (*p* < 0.05, Bonferroni-adjusted). † Significant difference between CAFcap and CAFrinse within the same time point (*p* < 0.05, Bonferroni-adjusted). ^a^ Significant difference relative to the 08:00 value within the same condition (*p* < 0.05, Bonferroni-adjusted). ^b^ Significant difference relative to the 12:00 value within the same condition (*p* < 0.05, Bonferroni-adjusted).

## Data Availability

The data supporting the findings of this study are available from the corresponding authors upon reasonable request due to privacy and ethical restrictions regarding the sensitive nature of the participant data.
